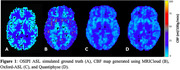# Towards Reproducible and Resource‐Efficient Perfusion Imaging Analysis for African Dementia Imaging Research

**DOI:** 10.1002/alz70856_104135

**Published:** 2025-12-26

**Authors:** Channelle Tham, Harrison Aduluwa, Ethan Draper, Jasmine Cakmak, Alfonso Fajardo, Oluwateniola Akinwale, Kesavi Kanagasabai, Philip Nkwam, Olusola Aremu, Cynthia Isabel Smith, Nsiah Donkor Anita, Issac Tigbee, Charity Umoren, Cristian Montalba, Kelvin Murithi, Confidence Raymond, Udunna Anazodo, Abdalla Z Mohamed

**Affiliations:** ^1^ Radboud University, Nijmegen, Netherlands; ^2^ Montreal Neurological Institute, McGill University, Montreal, QC, Canada; ^3^ Montreal Neurological Institute, Montreal, QC, Canada; ^4^ Integrated Program in Neurosciences, McGill University, Montréal, QC, Canada; ^5^ Johns Hopkins University, Baltimore, MD, USA; ^6^ Department of Medical Biophysics, Western University, London, ON, Canada; ^7^ University of Lagos, Lagos, Nigeria; ^8^ Department of Medicine, Lagos State University, Lagos, Nigeria; ^9^ Brain and Mind Institute, Aga Khan University, Nairobi, Nairobi, Kenya; ^10^ Department Of Medical Imaging Technology, University For Development Studies, Tamale, Ghana; ^11^ Medical Artificial Intelligence Laboratory, Crestview Radiology Ltd, Lagos, Nigeria; ^12^ Biomedical Imaging Center, Pontificia Universidad Católica de Chile, Santiago, Santiago, Chile; ^13^ Sonar Imaging Centre, Nairobi, Kenya; ^14^ Department of Biomedical Engineering, McGill University, Montreal, QC, Canada; ^15^ Biomedical Egineering, McGill University, Montreal, ON, Canada; ^16^ Medical Artificial Intelligence (MAI) Laboratory, Crestview Radiology Limited, Lagos, Nigeria; ^17^ Center for Brain and Health, New York University‐ Abu Dhabi, Abu Dhabi, United Arab Emirates

## Abstract

**Background:**

Arterial spin labelling (ASL) is an established magnetic resonance imaging (MRI) technique for non‐invasive assessment of cerebral blood flow (CBF). However, the lack of expertise and the costly computational resources required to analyze ASL data are major barriers to its use in resource‐constrained settings (RCS), particularly in Africa. ASL‐MRICloud was recently introduced as the only cloud‐based open‐source option for ASL processing that requires no installation on local computers, making it suited for ASL analysis in RCS.

**Methods:**

In this work, we implemented ASL‐MRICloud in Google Colab to perform data analysis at scale and minimal cost, with the aim of enhancing population studies reproducibility in RCS. This work was performed as a training exercise of the CONNExIN (COmprehensive Neuroimaging aNalysis Experience In resource‐constraiNed Settings) Program, a neuroimage analysis training program for African researchers. A team of CONNExIN participants leveraged the Open Science Initiative for Perfusion Imaging ASL (OSIPI‐ASL) MRI Challenge dataset (*n* = 10) to test the implemented ASL‐MRICloud Google Colab. The pipeline included a data preparation step for data conversion and generation of parameter files to be used for data processing. ASL processing and CBF quantification were then performed using automated steps in ASL‐MRICloud to generate whole‐brain CBF maps and extract preset regional values. The pipeline was validated by comparing the CBF maps and regional values to their ground‐truth, ASL‐MRICloud developer analysis, and results from other established ASL processing tools (Oxford ASL and Quantiphyse). The Google Colab implementation was provided to four CONNExIN teams to replicate the challenge data processing and analyze the dataset of 75 subjects from the PREVENT‐AD (PResymptomatic EValuation of Experimental or Novel Treatments for AD) study.

**Results:**

Preliminary results from the simulated OSIPI‐ASL dataset generated from our pipeline are shown in Figure 1. The pipeline, documentation, and results of the analysis from the four teams will be made publicly available on Protocol.io.

**Conclusion:**

Using simulated (OSIPI‐ASL) and real‐world (PREVENT‐AD) data, we aim to assess the feasibility of a resource‐efficient image processing tool for reproducible ASL data analysis. Once implemented, we will share our approach for wider use in RCS to enable inclusive and reproducible imaging research.